# Loss of mature D1 leads to compromised CP43 assembly in *Arabidopsis thaliana*

**DOI:** 10.1186/s12870-021-02888-9

**Published:** 2021-02-20

**Authors:** Yafei Shi, Yufen Che, Yukun Wang, Sheng Luan, Xin Hou

**Affiliations:** 1 State Key Laboratory of Hybrid Rice, College of Life Sciences, Wuhan University, Wuhan, 430072 China; 2 Department of Plant and Microbial Biology, University of California, Berkeley, CA 94720 USA

**Keywords:** Precursor D1, Photosystem II, PSII supercomplexes, Reaction center, CtpA, *Arabidopsis thaliana*

## Abstract

**Background:**

Photosystem II (PSII) is a highly conserved integral-membrane multi-subunit pigment-protein complex. The proteins, pigments, lipids, and ions in PSII need to be assembled precisely to ensure a proper PSII biogenesis. D1 is the main subunit of PSII core reaction center (RC), and is usually synthesized as a precursor D1. D1 maturation by the C-terminal processing protease CtpA is essential for PSII assembly. However, the detailed mechanism about how D1 maturation affects PSII assembly is not clearly elucidated so far. In this study, *Arabidopsis thaliana CtpA* mutant *(atctpa:* SALK_056011*)*, which lacks the D1 mature process, was used to investigate the function of this process on PSII assembly in more details.

**Results:**

Without the C-terminal processing of precursor D1, PSII assembly, including PSII monomer, dimer, especially PSII supercomplexes (PSII SCs), was largely compromised as reported previously. Western blotting following the BN-2D-SDS PAGE revealed that although the assembly of PSII core proteins D2, CP43 and CP47 was affected by the loss of D1 mature process, the incorporation of CP43 was affected the most, indicated by its most reduced assembly efficiency into PSII SCs. Furthermore, the slower growth of yeast cells which were co-transformed with pD1 and CP43, when compared with the ones co-transformed with mature D1 and CP43, approved the existence of D1 C-terminal tail hindered the interaction efficiency between D1 and CP43, indicating the physiological importance of D1 mature process on the PSII assembly and the healthy growth of the organisms.

**Conclusions:**

The knockout *Arabidopsis atctpa* mutant is a good material to study the unexpected link between D1 maturation and PSII SCs assembly. The loss of D1 maturation mainly affects the incorporation of PSII core protein CP43, an inner antenna binding protein, which functions in the association of LHCII complexes to PSII dimers during the formation of PSII SCs. Our findings here provide detailed supports of the role of D1 maturation during PSII SCs assembly in higher plants.

**Supplementary Information:**

The online version contains supplementary material available at 10.1186/s12870-021-02888-9.

## Background

Photosynthesis harnesses sunlight to assimilate carbon dioxide and produce the biomass that is essential for almost all life on earth. The initial step of photosynthesis is water-plastoquinone oxidoreduction catalyzed by PSII. PSII is a highly conserved integral-membrane multi-subunit pigment-protein complex found in cyanobacteria, algae, and plants. PSII components include core proteins, low-molecular-mass (LMM) proteins, extrinsic oxygen-evolving complex (OEC) proteins, and light-harvesting complex (LHC) proteins [1–6]. To ensure the proper PSII biogenesis, at least 20 different subunit proteins, as well as different pigments, lipids and ions need to be assembled precisely [7]. The De novo assembly of PSII in higher plants is as flows: the core “reaction center” (RC) complex which consists of D1, D2, PsbE, PsbF and PsbI is assembled at first. Subsequently, CP47 module is assembled with the attachment of the inner antenna protein CP47 and LMM proteins, such as PsbH, PsbM, PsbT, PsbR, to RC. Next, with the subsequential binding of CP43, OEC proteins and LMM proteins such as PsbK, PsbW, PsbZ, the RC47 complex transforms to PSII core monomer. Later, with the dimerization of PSII monomer and the attachment of LHCII, PSII-LHCII supercomplexes is formed [7–12].

D1 protein, which is encoded by the chloroplast gene *PsbA*, is a core subunit of PSII and involves in PSII photodamage and repair cycle. PSII repair is a protection mechanism of chloroplasts from damages caused by excessive illumination. During this process, the most frequently damaged D1 is degraded and replaced with a new copy to form a new RC [13]. Although a small *PsbA* gene family with three to four gene copies exists in cyanobacteria, D1 protein is encoded by a single *PsbA* gene in plastome [14, 15]. All photosynthetic organisms synthesize D1 protein as a precursor form (pD1) with a various-length of extensions at the C-terminal. The mature form of D1 is a prerequisite for Mn_4_CaO_5_ cluster formation and the binding of extrinsic proteins to PSII [16].

During the formation of the RC complex, the C-terminus extension of pD1 is processed by the C-terminal processing protease (CtpA) to generate mature D1. CtpA is a serine endopeptidase with a serine/lysine catalytic dyad [17–19]. In *Synechocystis PCC 6803*, three Ctp homologs (CtpA, CtpB, and CtpC) have been discovered and only CtpA can cleavage the pD1 C-terminal extension. *Synechocystis PCC 6803* mutant lacking pD1 C-terminal processing is more susceptible to photodamage [20, 21]. Consistent with *Synechocystis PCC 6803*, three putative CtpA homologues (At4g17740, At3g57680, and At5g46390) have also been found in *Arabidopsis*. Loss function of *CtpA* leads to compromised PSII activity and oxygen evolution [16, 22]. The mature form of D1 protein is absent, and the abundance of other PSII core proteins is reduced in *Arabidopsis atctpa* mutant which lacks the D1 maturation process [23]. Based on the divergence, the molecular function of D1 maturation needs to be further explored.

In this study, we isolated PSII complexes containing only pD1 from a *CtpA* null mutant of *Arabidopsis thaliana* and investigated the role of D1 maturation in the PSII assembly process in more details. Our result showed that loss of mature D1 protein led to a compromised assembly of PSII SCs, which was caused by the abnormal assembly of RC complexes, especially the subunit of CP43, revealing the distinct and important function of D1 mature process during PSII SCs assembly.

## Results

### Loss of CtpA results in the accumulation of pD1 in *Arabidopsis*

**Fig. 1:**
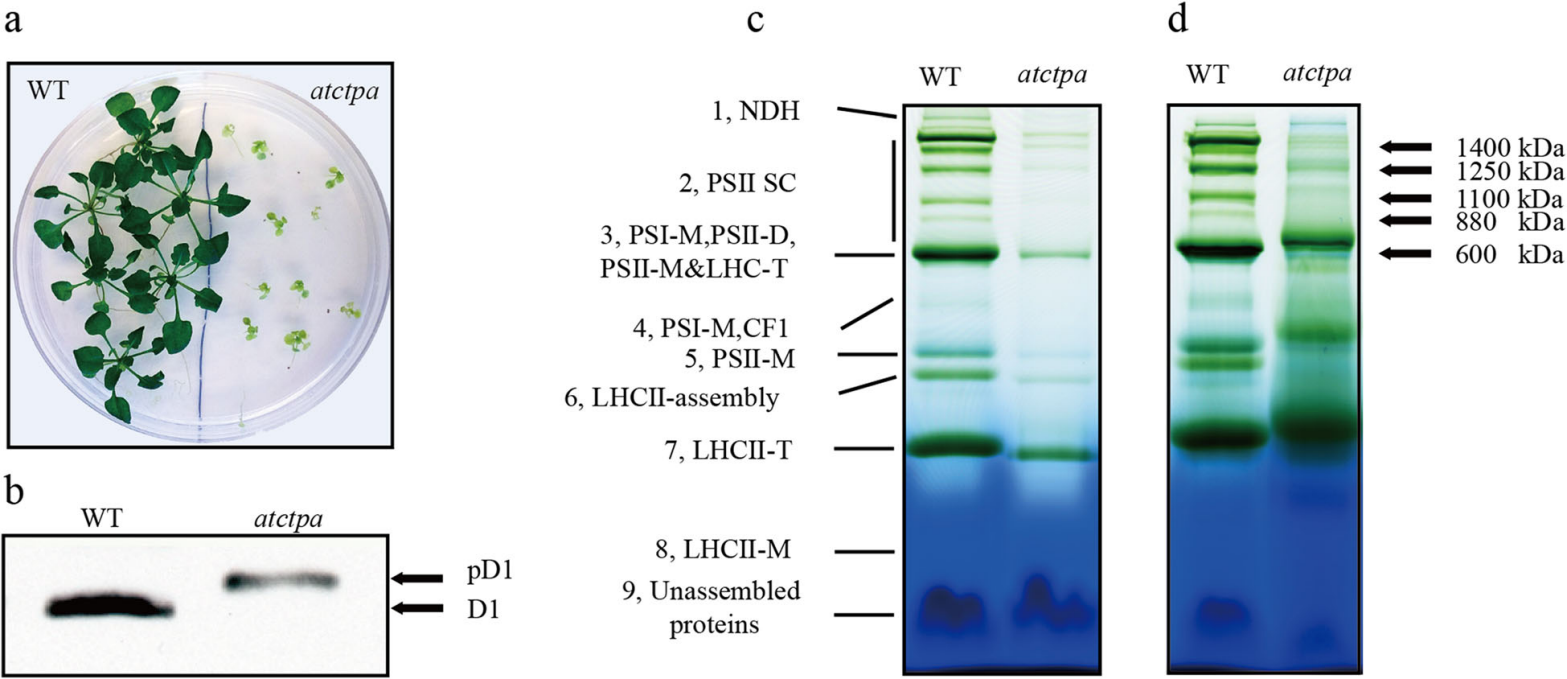
Characterization of *atctpa* mutant. **a** WT and *atctpa* were grown on 1/2 MS medium with 3.0% sucrose for 4 weeks under the conditions of 16 h light / 8 h dark cycle and 20 μmol photons m^− 2^ s^− 1^ light intensity during the light periods. **b** Immunoblot analysis of D1 protein. Thylakoid proteins were isolated from WT and *atctpa* mutant plants. Five μg chlorophyll was loaded and subsequently immunodetected with D1 antibody. **c, d** Blue native gel analysis (BN-PAGE) of thylakoid membrane complexes in WT and *atctpa* mutant based on an equal fresh weight **c** and an equal chlorophyll (chl) **d**. NDH, NADPH dehydrogenase complexes; PSII SCs, PSII-LHCII supercomplexes; PSI-M, PSI monomers; PSII-D, PSII dimers; PSII-M, PSII monomers; CF1: ATPase complex; LHCII-T, LHCII trimers; LHCII-M, LHCII monomers

Our earlier work had shown that pD1 protein cannot be processed into mature D1 protein without CtpA function [23]. To further study the physiological role of D1 mature process, the previously used *Arabidopsis* T-DNA insertion mutant *atctpa* (At4g17740, SALK_056011), which lacks the D1 mature process, was used in our current study. The 4-week-old *atctpa* mutants exhibited a wide range of defects in plant development under growth conditions when compared with WT, including a stunted growth and yellowish leaves (Fig. 1a). Next, we confirmed the form of D1 protein in *atctpa* mutant and WT by Western Blot. The results showed that only pD1 form was detected in *atctpa* mutant, while D1 protein existed in WT was the mature form (Fig. 1a). All these results were consistent with the previous report [23]. Based on this, we think *atctpa* mutant is an ideal material to study the role of D1 maturation in PSII assembly in *Arabidopsis*.

### The assembly of PSII SCs is aberrant without mature D1

The C-terminal processing of D1 protein is essential for PSII assembly in cyanobacteria and higher plant like *Arabidopsis* [17, 20, 21, 23, 24]; however, the mechanisms about how D1 mature process functions during PSII assembly, especially in higher plants, are not clearly elucidated yet. To investigate the D1 maturation function in more details, the assembly status of thylakoid membrane complexes with and without D1 mature process was checked by blue native gel analysis using WT and *atctpa* mutant, followed with a quantitative analysis. The result showed that, besides the assembly defects in PSII monomer and dimer, which was consistent with the former findings [17, 20, 24], a significant decrease in PSII SCs was found in *atctpa* mutant compared with WT, according to both analysis when either based on an equal fresh weigh (Fig. 1c) or an equal chlorophyll (Fig. 1d).

**Fig. 2:**
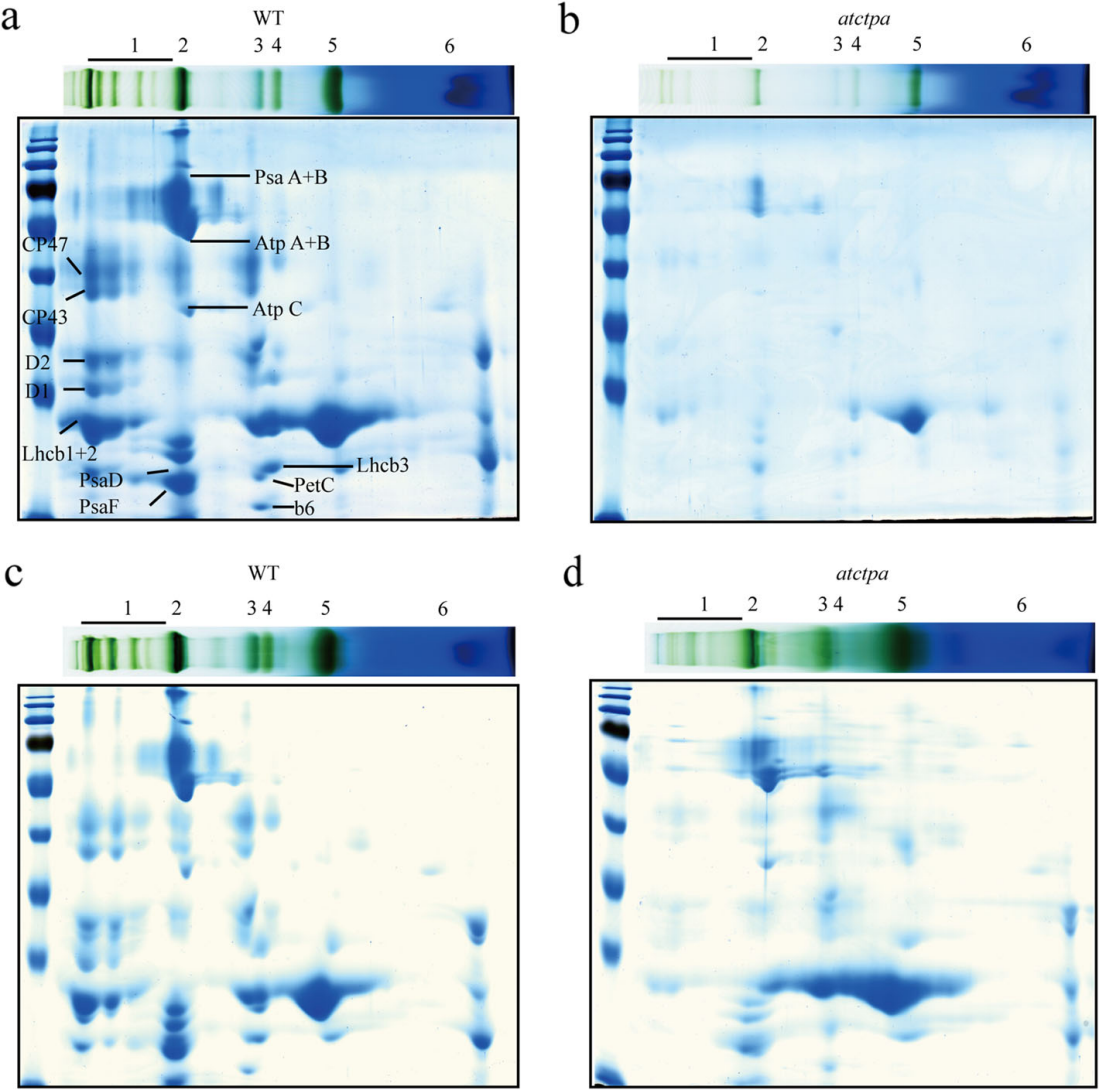
Thylakoid membrane complexes analysis in WT and *atctpa* mutant. **a, b** Coomassie blue staining of the 2D SDS-PAGE gels followed the BN-PAGE loaded with the same amount of fresh weight of WT **a** and *atctpa* **b**. **c, d** Coomassie blue staining of the 2D SDS-PAGE gels followed the BN-PAGE loaded with the 15 μg chl of WT **c** and *atctpa* **d**. Protein identification in Fg.2 was according to Hou et al. [25] and Fu et al. [26]. lane 1, PSII-SCs; lane 2, PSI-M, PSII-D, PSII-M&LHC-T; lane 3, PSII-M; lane 4, LHC assembly, PSII core lack CP43; lane 5, LHC-T and lane 6, LHC-M. The thylakoid complexes were labeled as indicated in the legend for Fig. 1

To further estimate the defects in PSII SCs assembly in lack of the D1 mature process, the blue native gel slices were subjected to the second-dimension electrophoresis by 2D SDS- PAGE and stained by commassie blue (CBB). Consistent with the defects in PSII SCs assembly as shown in Fig. 1c and d, the assembly efficiency of D1 (PsbA), D2 (PsbD), CP43 (PsbC), and CP47 (PsbB) into PSII SCs reduced dramatically in *atctpa* mutant compared with WT, when the analysis was conducted either based on an equal fresh weight (Fig. 2a, b) or an equal chlorophyll (Fig. 2c, d).

**Fig. 3:**
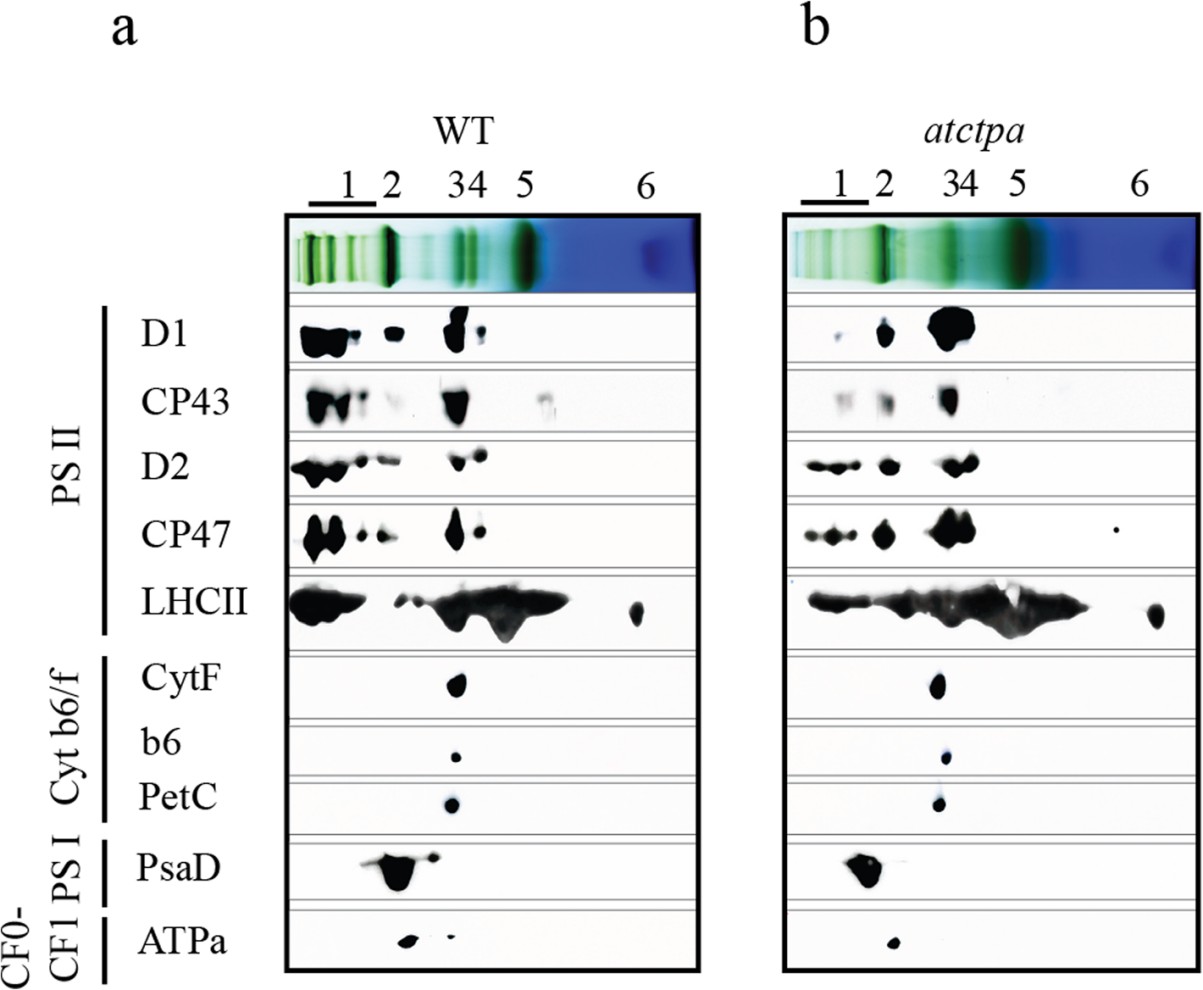
Assembly status of thylakoid membrane complex proteins in WT and *atctpa* mutant. **a, b** Thylakoid proteins (15 μg chl) of WT **a** and *atctpa* mutant **b** were separated by 2D BN/SDS-PAGE and further subjected to immunoblotting as indicated. Specific antibodies against D1, CP43, D2, CP47, LHCII, CytF, b6, PetC, PsaD and ATPα were used for immunodetection of the corresponding proteins, respectively. The thylakoid complexes were labeled as indicated in the legends for Figs. 1 and 2

To confirm the changes observed above, immunoblots following 2D-SDS-PAGE were performed. Consistently, the changes in the assembly efficiency of PSII main subunits were the same as shown in Fig. 2. As a control, the assembly of Cytochrome b_6_/f complex (Cyt b_6_/f), PSI and CF_o_-CF_1_ complex subunits remained unchanged according to the analysis done on the equal chlorophyll basis (Fig. 3a, b). The results were repeatable using plants grown in different greenhouses with the same growth environments (Fig. S1a, b and Tab. S1).

### Loss of mature D1 process disturbs PSII SCs assembly by compromising CP43 assembly

As we mentioned earlier, PSII SCs assembly is a well-organized and sequential process involving precise protein-protein interactions and the assistance of many auxiliary proteins [7]. To figure out the assembly pattern of each PSII core subunit and LHCII proteins with the loss of C-terminal processing of pD1, the immunoblots of 2D SDS-PAGE were further analyzed by ImageJ (https://imagej.nih.gov/ij/) (Table 1). The blue native gel analysis were divided into six sections: lane 1, PSII SCs; lane 2, PSI monomers (PSI-M), PSII dimers (PSII-D), and PSII monomer (PSII-M) binding with LHCII trimer (LHCII-T); lane 3, PSII-M and Cyt b_6_/f; lane 4, LHCII assembly, PSII core lack CP43; lane 5, LHCII-T; lane 6, LHCII monomer (LHCII-M). According to the result, pD1 could barely be assembled into PSII SCs without the C-terminal processing of pD1, and mainly accumulated in PSII-M and RC47 (with 49.63 and 30.42% distribution ratio in PSII-M and RC47, respectively) (Table 1). Interestingly, among the other PSII core proteins, only CP43 showed a similar assembly pattern as pD1, while D2 and CP47 could remain a certain level of assembly in PSII SCs, although the assembly efficiency was lower when compared with that in WT.

**Table 1:** The assembly pattern analysis of the main thylakoid membrane proteins in WT and *atctpa* checked in Fig. 3

Thylakoid proteins	Plants	1, PSII-SC	2, PSI –M, PSII-D, PSII-M &LHC-T	3, PSII-M, Cyt b6/f	4, LHC assembly, PSII core lack CP43	5, LHC-T	6, LHC-M
D1	WT	53.69%	10.19%	32.00%	4.12%	–	–
	*atctpa*	1.23%	18.71%	49.63%	30.42%	–	–
CP43	WT	61.83%	1.22%	36.95%	–	–	–
	*atctpa*	9.88%	23.98%	66.14%	–	–	–
D2	WT	70.21%	8.63%	11.80%	9.35%	–	–
	*atctpa*	34.56%	21.55%	23.90%	20.00%	–	–
CP47	WT	62.99%	7.76%	23.39%	5.86%	–	–
	*atctpa*	21.43%	22.26%	36.25%	20.06%	–	–
LHCII	WT	30.00%	5.45%	15.86%	23.19%	21.64%	3.85%
	*atctpa*	13.04%	16.42%	14.04%	18.91%	32.56%	5.03%
CytF	WT	–	–	100.00%	–	–	–
	*atctpa*	–	–	100.00%	–	–	–
b6	WT	–	–	100.00%	–	–	–
	*atctpa*	–	–	100.00%	–	–	–
PetC	WT	–	–	100.00%	–	–	–
	*atctpa*	–	–	100.00%	–	–	–
PsaD	WT	–	91.33%	8.67%	–	–	–
	*atctpa*	–	100.00%	–	–	–	–
ATPα	WT	–	89.28%	10.72%	–	–	–
	*atctpa*	–	100.00%	–	–	–	–

The assembly pattern analysis of the main thylakoid membrane proteins checked in Fig.3 by Image J. --, not detected

Accordingly, the assembly efficiency of LHCII showed a similar tendency as CP43, with less accumulation in PSII SCs, and more accumulation in LHCII-T. During the PSII de novo assembly, D1 combines to D2 first, then CP47 is inserted to form RC47 complex. It is only after the insertion of CP47, CP43 can be inserted in a right manner to form the PSII RC complex [7–10]. Taken together, the existence of C-terminal of pD1 mainly affected the assembly of CP43, which resulted in the lower assembly efficiency of LHCII proteins to form PSII SCs, since LHCII complexes mainly associate with PSII dimer complexes through antenna-binding proteins, for instance, CP43 [27].

### The loss of D1 maturation process hinders the interaction of D1 with D2 and CP43 indicated by the yeast-two hybrid analysis

**Fig. 4:**
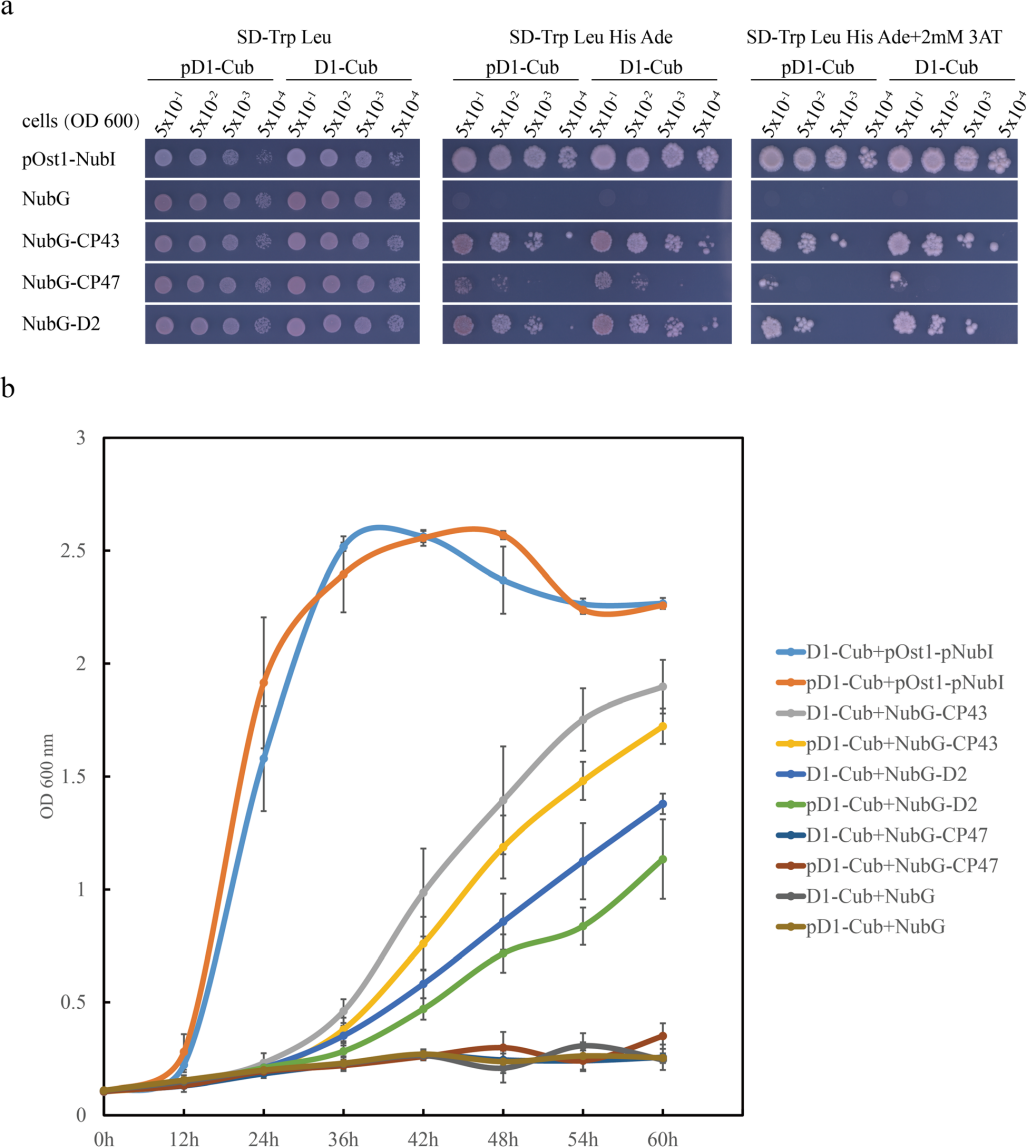
The C-terminal extension of pD1 hindered the interaction of D1 with RC complex proteins in yeast two-hybrid analysis. **a** Yeast two-hybrid analysis of the interactions between mature D1 / pD1 and RC complex proteins. Yeast cells expressing D1-Cub or pD1-Cub with various NubG-X (CP43, CP47 and D2) constructs were grown to logarithmic phase (OD_600_ = 0.5), and 5 μl portions of 1:10 serial dilutions were spotted on SD-Trp-Leu plates, and SD-Trp-Leu-His-Ade plates without or with 2 mM 3-AT, and incubated at 30 °C for 2 days. pDSL-Nx vector was used as the negative control, and NubI was used as the positive control. **b** Growth curves of yeast strains cultured in SD-Trp-Leu-His-Ade liquid medium containing 30 μg / ml kanamycine. Cells were grown at 30 °C with 200 rpm for the indicated period. Statistical analysis was shown in Tab. S2. Different letters indicated a significant difference among different values. Duncan’s multiple range test, *p* ≤ 0.05, *n* = 6

To confirm the hypothesis we mentioned above, we employed the mating-based yeast split ubiquitin system which can identify protein-protein interactions between integral (thylakoid) membrane proteins [28] to check whether the interaction efficiency between D1 and other PSII core subunits was affected or not with the C-terminal. Full-length mature D1 (amino acids 1–344) and pD1 (amino acids 1–353) were used as bait and Full-length D2, CP43 and CP47 were used as prey. According to the result, although both mature D1 and pD1 could interact with CP43 and D2, the interaction efficiency between D1 / pD1 and D2 was lower than that with CP43, indicating that the binding of mature D1 / pD1 with CP43 was stronger than it with D2 (Fig. 4a). In addition, the yeast cells expressing mature D1 and CP43 / D2 grew faster than the ones expressing pD1 and CP43 / D2 on the Synthetic Defined (SD) medium lacking Trp, Leu, His, and Adenine (SD-Trp Leu His Ade) (Fig. 4a), indicating that the existence of the C-terminal of pD1 hindered the interaction of D1 with CP43 / D2. In order to identify the differences among the transformed yeast cells with the above mentioned genes more clearly, we next monitored the growth curves of transformed yeast cells as shown in Fig. 4b and Tab. S2. Indeed, the growth rate of transformed yeast cells with pD1 was slower than the ones transformed with mature D1, confirming the affected interaction efficiency without the D1 maturation process.

## Discussion

In cyanobacterium *Synechocystus 6803*, the removal of C-terminal tail of pD1 is a perquisite for the assembly of manganese cluster, which is essential for a fully functional PSII complex [10, 17, 29]. It was also pointed out that D1 mature process is necessary for the binding of PSII extrinsic proteins into PSII [16]. However, the defects caused by the loss of D1 maturation process in higher plants were not exactly the same as mentioned above. In *atctpa* mutant whose D1 mature process is lost, although the water-oxidizing manganese cluster became dysfunctional indicated by the sever decrease in oxygen evolution, the binding of outer proteins (PsbO, PsbP and PsbQ) were not affected much [23]. The most obvious change was the decrease of the assembly of PSII SCs (Figs. 1, 2), which does not exist in cyanobacterium *Synechocystis* sp. PCC 6803.

**Fig. 5:**
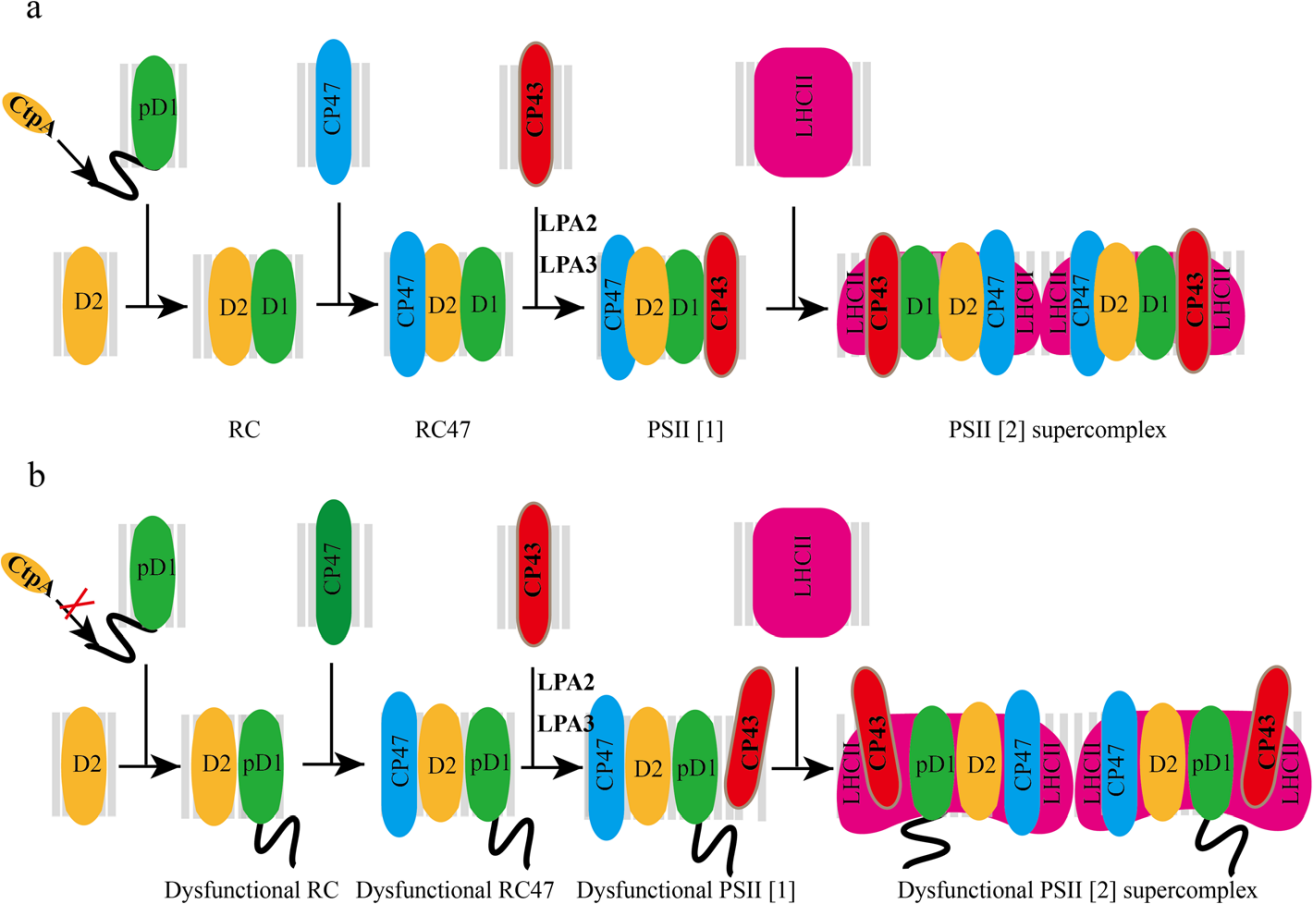
A proposed functional scheme of C-terminal processing of pD1 during PSII assembly. **a** The normal PSII assembly in higher plants. **b** The loss of C-terminal processing of pD1 causes a less efficient/stable assembly of CP43 during the PSII assembly in higher plants

PSII is a multi-subunit complex, and its assembly is a precisely controlled and sequential process. As shown in Fig. 5, pD1 interacts with PSII initiation complexes which contain D2 to form PSII minimal RC complexes. Later, CP47 is recruited to form PSII RC47 complexes. Then CP43 is assembled to generate PSII monomer, and PSII dimer is formed with the dimerization of PSII monomer [7]. In vascular plants, the PSII main peripheral antenna proteins form LHCII trimers, which bind to PSII dimers with the aid of minor LHCII species (CP24, CP26 and CP29) in either strongly-bound or moderately-bound manner through their associations with PSII core proteins CP43 / CP47 [24, 30, 31].

It has pointed out that pD1 can interact with D2-cyt b559 complexes to form D1-D2 complex, whereas CP47 and CP43 are incorporated into the D1-D2 complex after the C-terminal processing of pD1 [12]. Consistently, here, we also found that the efficient assembly of D2 was not affected much by the existence of pD1. However, the efficient assembly of CP43 was obviously affected. Interestingly, although the insertion of CP43 only occurs after the proper assembly of CP47, the assembly of CP47 was not much affected by the loss of D1 maturation (Fig. 3, Table 1 and Fig. S1, Tab. S1). This is explainable because that CP47 locates closely to D2, while CP43 locates closely to D1 according to the PSII SCs structure [27, 32–34]. Furthermore, Fig. 4 confirmed the lower interaction efficiency of pD1 and CP43 compared with that between mature D1 and CP43, indicated by the slower growth rate of yeast cells co-transformed with pD1 and CP43, when compared with the ones co-transformed with mature D1 and CP43. Overall, these data suggest that the defective assembly of PSII SCs without D1 maturation is mainly caused by the disturbed efficient assembly of CP43, which affects the subsequent PSII SCs assembly process consequently. For instance, the defected association of LHCII to PSII dimer (Fig. 3, Table 1 and Fig. S1, Tab. S1), whose association with PSII mainly involves in the inner antenna proteins, like CP43 in higher plants [27, 35]. Additionally, as a secondary effect of PSII deficiency, PSI components and ATPα levels were reduced (Fig. 3).

Much information has been obtained concerning the binding of CP43 to PSII RC complex. Two well-studied CP43 assembly factors are Low PSII Accumulation2 (LPA2) and Low PSII Accumulation3 (LPA3) [36, 37]. LPA2 is a small intrinsic thylakoid membrane protein, while LPA3 has no transmembrane domain. Although they are not homologous, they are identified to assist incorporation of CP43 into PSII via the interaction with the thylakoid membrane protein Albino 3 (ALB3), a cpSRP translocase. Our results here showed that AtCtpA, the C-terminal processing protease of pD1, also plays an important function of the effectively incorporation of CP43 into PSII SCs. A proposed D1 mature process functional scheme summarized from the current work was illustrated in Fig. 5: pD1 is incorporated into D2-cyt b559 complex to form RC complex which consists of pD1, D2, PsbE, PsbF and PsbI. During the formation of RC complex, pD1 is processed at its C-terminus extension by AtCtpA to yield mature D1. Without D1 mature process, although the corporation of inner-antenna protein CP47 is not affected much, the formed RC47 complex is dysfunctional. Next, CP43 is inserted into the dysfunctional RC47 complex with a much lower assembly efficiency to form the dysfunctional monomeric PSII (PSII [1]). Finally, the dysfunctional PSII [1] forms the dysfunctional dimer PSII (PSII [2]). During this process, the C-terminus extension of pD1 would change the spatial conformation of pD1-RC47 complex, which affects the correct assembly of CP43 and the subsequent assembly of other subunits. Besides this, the associations of PSII SCs subunits would become loosely due to the existence of pD1, which result in an easier detachment of certain subunits from the PSII SCs during the thylakoid membrane isolation and solubilization process in the presence of detergent, which consists with the decrease of the PSII SCs in *atctpa* mutant as we have found (Figs. 2, 3, 4 and Table 1).

## Conclusions

By studying the PSII SCs assembly in more details using the *atctpa* mutant which lacks the C-terminal processing process of pD1, we found that the defects in PSII SCs assembly caused by the loss of D1 maturation mainly lie in the deficient incorporation of PSII core protein CP43, an inner antenna binding protein, which functions in the association of LHCII complexes to PSII dimers during the formation of PSII SCs. Our finding indicates the mechanism how D1 maturation process functions during PSII SCs assembly in higher plants.

## Methods

### Plant materials and growth conditions

*Arabidopsis* (Columbia-0) and the T-DNA insertion mutant line (SALK_056011, locus At4g17740) were obtained from the *Arabidopsis* Resource Center (Columbus, OH). Seedlings were grown on 1/2 MS medium containing 3.0% sucrose (pH 5.7) for 4 weeks under the conditions of 16 h light / 8 h dark cycle and 20 μmol photons m^− 2^ s^− 1^ light intensity during the light periods.

### Blue native PAGE and 2D SDS-PAGE

Chloroplasts were extracted from 50 wild-type and 50 *atctpa*-mutant plants. Blue native gel electrophoresis was performed as described previously [25, 38]. For 2D SDS-PAGE, the blue native gel lanes were excised with a razor blade and incubated in 2 × SDS sample buffer containing 2.5% (vol / vol) β-mercaptoethanol (β-ME) for 20 min at 75 °C, then for 20 min at 25 °C. Lanes with denatured proteins were placed on top of 12% SDS gels, then subjected to the second dimensional separation.

### Immunoblot analysis

For immunoblotting, protein samples were separated on 12% SDS gels and transferred to nitrocellulose membranes (BioTrace™ NT nitrocellulose, Mexico) followed by a western blot analysis. After blocking with 5% milk, the membranes were subsequently incubated with primary antibodies generated against the indicated proteins and detected using the Super Signal™ West Pico PLUS Chemiluminescent Substrate kit (Thermo Scientific, USA).

### Yeast two-hybrid assay and growth curves analysis

The yeast two-hybrid assay was performed using the Split Ubiquitin System (DUAL membrane, Dualsystems Biotech) as described previously [39, 40]. The mature D1 (amino acids 1–344) and pD1 (amino acids 1–353) were cloned into pCCW-STE vector (encoding the Cub-LexA-VP16 fragment) as the bait for interaction assay. CP43, CP47 and D2 were cloned into pDSL-Nx vector (encoding the NubG fragment) as the prey for the assay. Yeast strain NMY32 was co-transformed with the bait and prey constructs, respectively. The interactions were determined by the growth of yeast cells on agar plates with Synthetic Defined (SD) medium lacking Trp, Leu, His, and adenine (SD-Trp Leu His Ade, FunGenome) without or with 2 mM 3-amino-1, 2, 4-Triazole (3-AT). The growth curves of liquid cultured yeast cells were obtained by measuring the absorbance at 600 nM (OD_600_). Six colonies of each transformation were cultured in SD-Trp Leu His Ade medium containing 30 μg / ml kanamycine. The OD_600_ values were recorded at various time points. pDSL-Nx vector was used as the negative control, and NubI was used as the positive control.

### Statistical analysis

ImageJ (https://imagej.nih.gov/ij/) was employed to qualify the distribution ratio of PSII subunits among different subcomplexes.

## Supplementary Information

**Additional file 1: Figure S1.** Assembly status of thylakoid membrane complex proteins in WT and *atctpa* mutant. (a, b) Thylakoid proteins (15 μg chl) of WT (a) and *atctpa* mutant (b) were separated by 2D BN/SDS-PAGE and further subjected to immunoblotting as indicated. Specific antibodies against D1, CP43, D2, CP47, LHCII, CytF, b6, PetC, PsaD and ATPα were used for immunodetection of the corresponding proteins, respectively. **Tab. S1.** The assembly analysis of the main thylakoid membrane proteins in WT and *atctpa* as checked in Figure S1. The assembly analysis of the main thylakoid membrane proteins as checked in Figure S1. by Image J. --, not detected. **Tab. S2.** Statistical analysis of growth curves as checked in Fig. 4b. Different letters indicated a significant difference among different values. Duncan’s multiple range test, *p* ≤ 0.05, *n* = 6. **Tab. S3.** Primers used in this study. The red letters represent the digestion sites of enzymes.

## Data Availability

All data generated or analyzed in this study are included in this article and its supplementary materials. The datasets used and/or analysed during the current study are available from the corresponding author on reasonable request. Biological materials used in the present study are available from the corresponding author upon reasonable request.

## Abbreviations

LHCII: Light-harvesting complexes
LHCII-M: LHC monomers
LHCII-T: LHCII trimers
LMM: Low-molecular-mass subunits
OEC: Oxygen-evolving complex
PSI: Photosystem I
PSII: Photosystem II
PSII SCs: PSII supercomplexes
PSII-D: PSII dimers
PSII-M: PSII monomers
PSI-M: PSI monomers
RC: Reaction center complex
