# Supplementary material for: Loss of mature D1 leads to compromised CP43 assembly in Arabidopsis thaliana
Source: BMC Plant Biol. 2021 Feb 20;21:106. doi: 10.1186/s12870-021-02888-9 (PMC7896377; doi:10.1186/s12870-021-02888-9)
Supplement: Supplementary file 1 — Additional file 1: Figure S1. Assembly status of thylakoid membrane complex proteins in WT and atctpa mutant. (a, b) Thylakoid proteins (15 μg chl) of WT (a) and atctpa mutant (b) were separated by 2D BN/SDS-PAGE and further subjected to immunoblotting as indicated. Specific antibodies against D1, CP43, D2, CP47, LHCII, CytF, b6, PetC, PsaD and ATPα were used for immunodetection of the corresponding proteins, respectively. Tab. S1. The assembly analysis of the main thylakoid membrane proteins in WT and atctpa as checked in Figure S1. The assembly analysis of the main thylakoid membrane proteins as checked in Figure S1. by Image J. --, not detected. Tab. S2. Statistical analysis of growth curves as checked in Fig. 4b. Different letters indicated a significant difference among different values. Duncan’s multiple range test, p ≤ 0.05, n = 6. Tab. S3. Primers used in this study. The red letters represent the digestion sites of enzymes. [file 12870_2021_2888_MOESM1_ESM.docx]

**Fig. S1.**


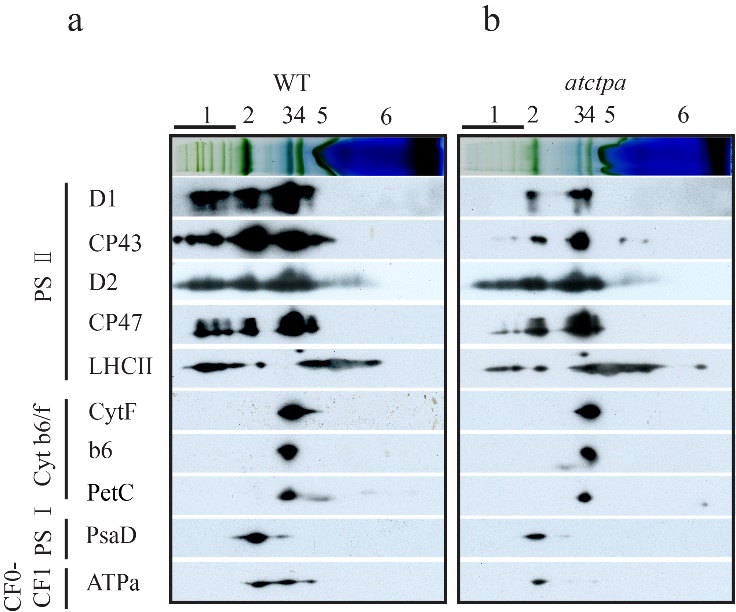


**Fig. S1. Assembly status of thylakoid membrane complex proteins in WT and *atctpa* mutant.**

**(a, b)** Thylakoid proteins (15 μg chl) of WT (a) and *atctpa* mutant (b) were separated by 2D BN-SDS PAGE and further subjected to immunoblotting as indicated. Specific antibodies against D1, CP43, D2, CP47, LHCII, CytF, b6, PetC, PsaD and ATPα were used for immunodetection of the corresponding proteins, respectively.

**Tab. S1.** **The assembly pattern analysis of the main thylakoid membrane proteins in WT and *atctpa* checked in Fig. S1.**

| Thylakoid proteins | Plants | 1, PSII-SC | 2, PSI –M, PSII-D, PSII-M &LHC-T | 3, PSII-M, Cyt b6/f | 4, LHC assembly, PSII core lack CP43 | 5, LHC-T | 6, LHC-M |
| --- | --- | --- | --- | --- | --- | --- | --- |
| D1 | WT | 36.21% | 20.16% | 30.80% | 12.83% | -- | -- |
|  | *atctpa* | 0.00% | 31.88% | 34.54% | 33.58% | -- | -- |
| CP43 | WT | 23.71% | 38.94% | 37.35% | -- | -- | -- |
|  | *atctpa* | 3.80% | 23.73% | 72.46% | -- | -- | -- |
| D2 | WT | 33.61% | 20.05% | 29.23% | 17.11% | -- | -- |
|  | *atctpa* | 27.68% | 22.48% | 30.80% | 19.04% | -- | -- |
| CP47 | WT | 29.08% | 21.65% | 33.97% | 15.29% | -- | -- |
|  | *atctpa* | 11.61% | 26.27% | 39.51% | 22.60% | -- | -- |
| LHCII | WT | 39.06% | 8.55% | 4.58% | 7.70% | 30.20% | 9.91% |
|  | *atctpa* | 21.63% | 7.64% | 6.03% | 13.09% | 45.62% | 5.98% |
| CytF | WT | -- | -- | 100.00% | -- | -- | -- |
|  | *atctpa* | -- | -- | 100.00% | -- | -- | -- |
| b6 | WT | -- | -- | 100.00% | -- | -- | -- |
|  | *atctpa* | -- | -- | 100.00% | -- | -- | -- |
| PetC | WT | -- | -- | 100.00% | -- | -- | -- |
|  | *atctpa* | -- | -- | 100.00% | -- | -- | -- |
| PsaD | WT | -- | 88.58% | 11.42% | -- | -- | -- |
|  | *atctpa* | -- | 92.59% | 7.41% | -- | -- | -- |
| ATPα | WT | -- | 84.13% | 15.87% | -- | -- | -- |
|  | *atctpa* | -- | 93.49% | 6.51% | -- | -- | -- |

The assembly pattern analysis of the main thylakoid membrane proteins checked in Fig. S1 by Image J. --, not detected.

**Tab. S2.** **Statistical analysis of growth curves in Fig. 4b.**

|  | 0h | | 12h | 24h | 36h | 42h | 48h | 54h | 60h |
| --- | --- | --- | --- | --- | --- | --- | --- | --- | --- |
| D1-Cub+  pOst1-pNubI | | 0.11±0.01 | 0.22±0.03 | 1.15±0.23^b^ | 2.52±0.02^a^ | 2.56±0.03^a^ | 2.37±0.15^a^ | 2.26±0.02^a^ | 2.27±0.02^a^ |
| pD1-Cub+  pOst1-pNubI | | 0.10±0.00 | 0.28±0.08 | 1.92±0.29^a^ | 2.40±0.17^b^ | 2.56±0.04^a^ | 2.57±0.02^a^ | 2.24±0.02^a^ | 2.26±0.01^a^ |
| D1-Cub+  NubG-CP43 | | 0.11±0.00 | 0.15±0.01 | 0.23±0.04^c^ | 0.16±0.05^c^ | 0.99±0.20^b^ | 1.39±0.24^b^ | 1.75±0.14^b^ | 1.90±0.12^b^ |
| pD1-Cub+  NubG-CP43 | | 0.10±0.00 | 0.13±0.01 | 0.20±0.02^c^ | 0.38±0.05^d^ | 0.76±0.12^c^ | 1.19±0.14^c^ | 1.48±0.08^c^ | 1.72±0.08^c^ |
| D1-Cub+  NubG-D2 | | 0.10±0.00 | 0.14±0.02 | 0.21±0.02^c^ | 0.35±0.04^d^ | 0.58±0.06^d^ | 0.86±0.12^d^ | 1.12±0.17^d^ | 1.38±0.05^d^ |
| pD1-Cub+  NubG-D2 | | 0.11±0.00 | 0.14±0.02 | 0.21±0.02^c^ | 0.28±0.04^e^ | 0.47±0.05^e^ | 0.72±0.08^e^ | 0.84±0.05^e^ | 1.13±0.18^e^ |
| D1-Cub+  NubG-CP47 | | 0.11±0.01 | 0.13±0.03 | 0.18±0.02^c^ | 0.23±0.03^e^ | 0.27±0.02^f^ | 0.24±0.04^f^ | 0.24±0.05^f^ | 0.26±0.06^f^ |
| pD1-Cub+  NubG-CP47 | | 0.11±0.01 | 0.13±0.01 | 0.19±0.01^c^ | 0.22±0.02^e^ | 0.26±0.02^f^ | 0.30±0.07^f^ | 0.24±0.05^f^ | 0.35±0.06^g^ |
| D1-Cub+  NubG | | 0.11±0.01 | 0.15±0.03 | 0.20±0.01^c^ | 0.23±0.03^e^ | 0.26±0.02^f^ | 0.21±0.06^f^ | 0.31±0.06^f^ | 0.24±0.01^g^ |
| pD1-Cub+  NubG | | 0.11±0.00 | 0.15±0.01 | 0.20±0.02^c^ | 0.23±0.01^e^ | 0.27±0.02^f^ | 0.24±0.05^f^ | 0.26±0.06^f^ | 0.25±0.02^g^ |

Different letters indicated a significant difference at 0.05 level (Duncan’s multiple range test) at the same time, n=6.

**Tab. S3. Primers used in this study.**

| Gene | sequences |
| --- | --- |
| D1-F | ATTAACAAGGCCATTACGGCCATGACTGCAATTTTAGAGAGACGC |
| D1-R | AACTGATTGGCCGAGGCGGCCCCAGCTAGGTCTAGAGGGAAGTTGTGA |
| pD1-F | ATTAACAAGGCCATTACGGCCATGACTGCAATTTTAGAGAGACGC |
| pD1-R | AACTGATTGGCCGAGGCGGCCCCTCCATTTGTAGATGGAGCCTCA |
| D2-F | ATTAACAAGGCCATTACGGCCATGACTATAGCCCTTGGTAAATTTACCA |
| D2-R | AACTGATTGGCCGAGGCGGCCTTAAAGAGCGTTTCCACGTGGTAG |
| CP43-F | ATTAACAAGGCCATTACGGCCATGAAAACCTTATATTCCCTGAGGAG |
| CP43-R | AACTGATTGGCCGAGGCGGCCTTAGTTAAGAGGAGTCATGGAAAGAACA |
| CP47-F | ATTAACAAGGCCATTACGGCCATGGGTTTGCCTTGGTATCGTG |
| CP47-R | AACTGATTGGCCGAGGCGGCCTCAGACTGCTTGTCGTTTTGTAGTTG |

The red letter represents the site of enzyme digestion.
